# The clinical impact of Lumacaftor-Ivacaftor on structural lung disease and lung function in children aged 6–11 with cystic fibrosis in a real-world setting

**DOI:** 10.1186/s12931-023-02497-0

**Published:** 2023-08-11

**Authors:** Paul McNally, Barry Linnane, Michael Williamson, Basil Elnazir, Christopher Short, Clare Saunders, Laura Kirwan, Rea David, Mariette P. C. Kemner-Van de Corput, Harm A.W.M. Tiddens, Jane C Davies, Des W Cox

**Affiliations:** 1 Respiratory Department, Children’s Health Ireland, Crumlin, Dublin, Ireland; 2 RCSI University of Medicine and Health Sciences, Dublin, Ireland; 3 University of Limerick School of Medicine, Limerick, Ireland; 4 Trinity College, Dublin, Ireland; 5 NHLI, Imperial College, London, UK; 6 Royal Brompton and Harefield Hospitals, Guy’s and St Thomas’ Trust, London, UK; 7 Cystic Fibrosis Registry of Ireland, Dublin, Ireland; 8 Department of Paediatric Pulmonology and Allergology, Department of Radiology and Nuclear Medicine, Erasmus Medical Centre - Sophia Children’s Hospital, Rotterdam, The Netherlands; 9 University College Dublin, Dublin, Ireland

**Keywords:** Lung clearance index, PRAGMA CF scores, Children, Real-world studies

## Abstract

**Background:**

Data from clinical trials of lumacaftor-ivacaftor (LUM-IVA) demonstrate improvements in lung clearance index (LCI) but not in FEV_1_ in children with Cystic Fibrosis (CF) aged 6–11 years and homozygous for the Phe508del mutation. It is not known whether LUM/IVA use in children can impact the progression of structural lung disease. We sought to determine the real-world impact of LUM/IVA on lung structure and function in children aged 6–11 years.

**Methods:**

This real-world observational cohort study was conducted across four paediatric sites in Ireland over 24-months using spirometry-controlled CT scores and LCI as primary outcome measures. Children commencing LUM-/IVA as part of routine care were included. CT scans were manually scored with the PRAGMA CF scoring system and analysed using the automated bronchus-artery (BA) method. Secondary outcome measures included rate of change of ppFEV_1_, nutritional indices and exacerbations requiring hospitalisation.

**Results:**

Seventy-one participants were recruited to the study, 31 of whom had spirometry-controlled CT performed at baseline, and after one year and two years of LUM/IVA treatment. At two years there was a reduction from baseline in trapped air scores (0.13 to 0.07, p = 0.016), but an increase from baseline in the % bronchiectasis score (0.84 to 1.23, p = 0.007). There was no change in overall % disease score (2.78 to 2.25, p = 0.138). Airway lumen to pulmonary artery ratios (A_lumen_A ratio) were abnormal at baseline and worsened over the course of the study. In 28 participants, the mean annual change from baseline LCI_2.5_ (-0.055 (-0.61 to 0.50), p = 0.85) measurements over two years were not significant. Improvements from baseline in weight (0.10 (0.06 to 0.15, p < 0.0001), height (0.05 (0.02 to 0.09), p = 0.002) and BMI (0.09 (0.03 to 0.15) p = 0.005) z-scores were seen with LUM/IVA treatment. The mean annual change from baseline ppFEV_1_ (-2.45 (-4.44 to 2.54), p = 0.66) measurements over two years were not significant.

**Conclusion:**

In a real-world setting, the use of LUM/IVA over two years in children with CF aged 6–11 resulted in improvements in air trapping on CT but worsening in bronchiectasis scores. Our results suggest that LUM/IVA use in this age group improves air trapping but does not prevent progression of bronchiectasis over two years of treatment.

**Supplementary Information:**

The online version contains supplementary material available at 10.1186/s12931-023-02497-0.

## Introduction

Deficient cystic fibrosis transmembrane conductance regulator (CFTR) activity in people with cystic fibrosis (CF) results in chronic airway infection and neutrophilic inflammation [1], leading to airway wall injury and bronchiectasis [2]. CF lung disease begins in early childhood [3] and despite having normal lung function as measured by spirometry, structural lung damage in the form of bronchiectasis is commonly identified on computerised tomography (CT) [4]. By the age of six years, one third of children with CF have structural lung changes identified by CT [5]. Prevention of the development or worsening of bronchiectasis is a key therapeutic goal in the care of children with CF.

Phe508del is the commonest CF-causing mutation [6]. The development of CFTR modulator treatments has been a significant breakthrough in CF care. Lumacaftor/ivacaftor (LUM/IVA), the first modulator combination targeting Phe508del, has been shown in clinical trials to improve lung function (measured by percent predicted Forced Expiratory Volume in 1 s (ppFEV_1_)), reduce pulmonary exacerbation rate and improve body mass index (BMI) in people with CF ≥ 12 years of age and homozygous for Phe508del mutation [7]. Subsequent real-world data from observational studies on the impact of LUM/IVA on this CF population demonstrated similar findings [8].

To date, only a limited number of clinical trials have used chest CT as an outcome measure [9]. A uniform approach to scanner standardisation, breathing manoeuvres and image reconstruction should be optimised when adopting chest CT as an outcome measure in research studies [9] and this can be challenging. Lung volumes obtained without prior education and coaching during the acquisition result in suboptimal lung volume levels and can lead to respiratory motion artefact [10, 11]. Spirometry-controlled CTs allow for a standardised lung volume during imaging, reduce artefacts, make for sensitive detection of CF-related lung damage and allow for better comparison between scans [12]. PRAGMA-CF, a recently developed CF specific CT scoring system, has been identified as a useful outcome measure to study the effect of newer disease modifying drugs in CF on lung structure [13, 14]. A recently published international randomised controlled study on 116 children with CF aged 4–6 years adopted the PRAGMA CF %Disease as the primary outcome measure and reported improvements in structural lung disease with inhaled hypertonic saline compared with isotonic saline [14]. A key feature of bronchiectasis is the abnormal ratio of bronchus dimensions to pulmonary artery diameter (BA ratio). Previous studies have demonstrated that BA ratios increase progressively with each airway generation on volume-controlled CT scans in people with CF but remain unchanged in controls [15]. An increase in BA ratios over time correlates with increased structural lung disease as measured using PRAGMA-CF [16, 17]. These increases in BA dimensions are more easily missed on routine CTs when they are not spirometry controlled [15].

Although ppFEV_1_ has historically been used as a primary outcome measure in clinical trials in adults and adolescents with CF, it is a less suitable modality to study younger children with relatively well-preserved ppFEV_1_. Lung clearance index (LCI), derived from multiple breath washout (MBW) has been identified as a more sensitive measure of early lung disease in children with CF when compared with ppFEV_1_ [18]. MBW can be easily performed in children and repeated over time. LCI can assist in identifying those who may benefit from earlier intervention [19]. In the original clinical trial of LUM/IVA in 103 children aged 6–11 and homozygous for Phe508del, no improvement in ppFEV_1_ was demonstrated but a significant improvement in LCI was seen [20]. Improvements in other parameters such as nutritional indices and health-related quality of life scores were also identified in this trial and the findings were sustained for up to 120 weeks [21].

One of the key goals of respiratory management in people with CF is to slow the progression of structural lung damage seen with untreated disease, and by extension, prevent disease occurring in those yet to develop it. Previous work has demonstrated a significant correlation between spirometry controlled CT scores and LCI measurements in school aged children with CF [22]. Our hypothesis was that the introduction of LUM/IVA in children with CF aged 6–11 years would result in improvements in PRAGMA CT sores and LCI measurements in a real-world setting. The aim of this study (CFORMS – Children’s Follow-up Orkambi Real-world MBW Study) was to assess the clinical impact of LUM/IVA treatment on children aged 6–11 years of age. Our objectives in this study were to determine firstly whether LUM/IVA could prevent the progression of bronchiectasis over two years, and secondly, whether LUM/IVA was associated with improvements in ventilation inhomogeneity in a real world setting in children with CF.

## Methods

This was a two-year real-world cohort study of LUM/IVA at four paediatric sites in Ireland involving children with CF aged 6–11 years and homozygous for the Phe508del mutation. Participants were recruited prior to commencing clinically prescribed LUM/IVA. Local Research Ethics Committees approved the study and informed consent and assent was obtained.

Children with clinically unstable CF at the time of recruitment or those involved in clinical trials of CFTR modulators were excluded from the study. Clinical data was collected and managed in collaboration with the CF Registry Ireland (CFRI) from one year before and two years after initiation of LUM/IVA. Primary outcome measures were mean change from baseline per annum in spirometry-controlled CT scores and LCI_2.5_ averaged over two years. Secondary outcome measures were change from baseline in ppFEV_1_, nutritional indices and exacerbations requiring hospital admissions.

Spirometry controlled chest CT scans were performed at baseline and then annually for two years in a subgroup of study participants at one site (Children’s Health Ireland (CHI), Crumlin). All staff involved in conducting spirometry controlled CTs were certified by the ECFS CTN lung imaging core facility (LungAnalysis), at Erasmus Medical Centre, Rotterdam. Spirometry controlled chest CT scanning protocol used the NDD EasyOne® portable spirometer (NDD Medical Technologies Inc, USA) as outlined previously [23]. CT scans were pseudonymised and sent to LungAnalysis for PRAGMA CF scoring as detailed in Rosenow et al. 2015 [24]. Intra-observer and inter-observer reliability for PRAGMA-CF scores were assessed for each CT scan performed. An ICC greater than 0·8 was rated as excellent, 0·6–0·8 was good, 0·4–0·6 was moderate, and lower than 0·4 was poor. All CTs were scored in random order by a certified and experienced observer. Structural lung disease was further evaluated using a fully automated system to measure airway and artery dimensions, LungQ v2.21, an artificial intelligence driven software (Thirona, Nijmegen), and validated against the manual PRAGMA CF scoring method as previously described [15]. Inner (B_in_) and outer (B_out_) bronchus diameters were divided by artery diameter to calculate B_in_/A- and B_out_/A-ratio and wall thickness (WT) was divided by artery diameter to calculate B_WT_/A-ratio [17]. Airway generation starting at the segmental bronchus (G0) and segmental generation were determined for every BA-pair [15]. Adherence was assessed using medication possession ratio based on submitted pharmacy records. Hospital admission data was collected from medical records.

The study’s intention was to perform multiple breath washout (MBW) testing at baseline and then six-monthly for 24 months (4 follow up measures). However, the collection of follow up LCI measurements was significantly hindered due to the COVID-19 pandemic. As a result, only a limited number of LCI measurements were taken on subjects (Table E1). Participants were included in the analysis if they had a LCI measurement taken within three months of initiation of LUM/IVA and had at least one follow-up measurement taken post initiation. Each measurement was taken when participants were clinically stable.

MBW operators were certified by the European CF Society (ECFS) Clinical Trials Network (ECFS-CTN) LCI core facility at RBHT, and all tests were centrally over-read. MBW was performed using the Exhalyzer®D (Ecomedics AG, Switzerland, Spiroware software version 3.1.6) in accordance with the testing procedure detailed by the 2013 ERS/ATS consensus statement [25]. MBW tests were analysed using Spiroware version 3.2.1 in line with the European Cystic Fibrosis Society (ECFS) LCI core facility standards. LCI_2.5_ results were expressed as the means of all technically acceptable trial results, with a minimum of two acceptable trials required [26].

### Statistical analysis

Each of the continuous responses (LCI_2.5_, ppFEV_1_, BMI z-score, height z-score and weight z-score) were analysed using random coefficients mixed models, with fixed and random intercepts and slopes. As measurements were taken at unevenly spaced time points due to the COVID-19 pandemic, time was included in the model as a continuous variable based on study day. Study day was defined as the number of days pre/post initiation of LUM/IVA therapy. The intercepts give the mean at initiation of therapy (baseline). For participants, an interaction with time period indicator variable was included in the model, to compare the slope over time before and after initiation of LUM/IVA therapy. The number of hospitalisations per annum was compared in the period before and after index date using a Poisson regression repeated measures model including time period (before/after initiation of LUM/IVA therapy) and their interaction. The CT parameters (% Bronchiectasis, %Mucus plugging, % Disease and % Trapped Air) were also analysed using random coefficients mixed models, with fixed and random intercepts and slopes. The BA parameters were analysed using generalised linear mixed models with time, generation and their interaction as fixed effects and generation and lobe as random effects. An autoregressive (AR (1)) structure was fitted to the variance covariance matrix for all repeated measures models. As all of the comparisons that were made were planned, no corrections were applied for multiple comparisons.

## Results

**Fig. 1:**
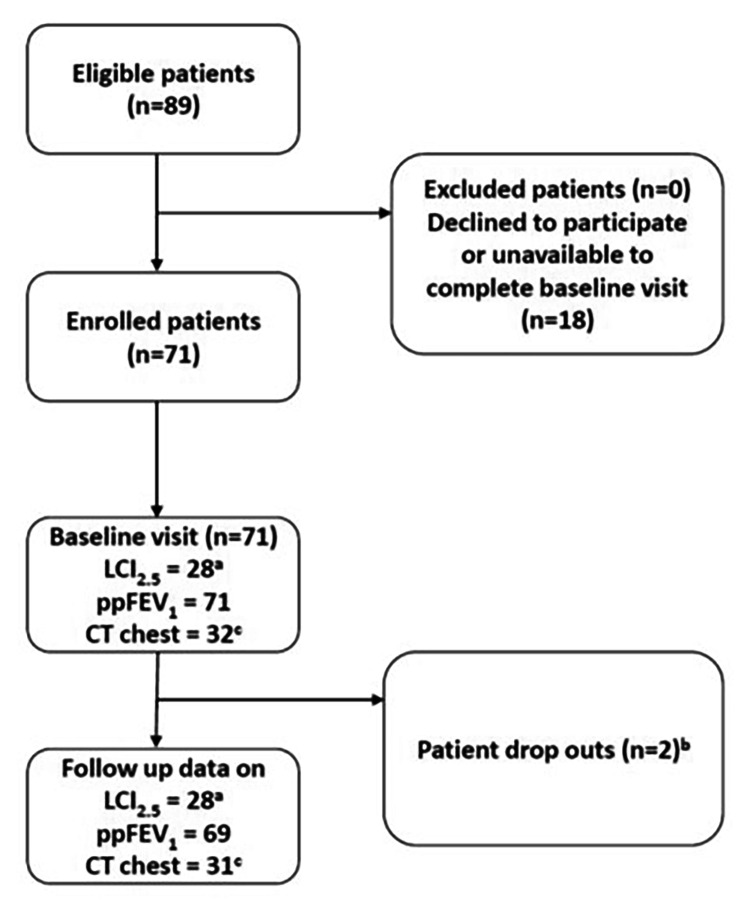
Participant flow diagram. **a**: Number of participants that had acceptable baseline and at least one follow up LCI_2.5_ measurement. **b**: Two participants left the study to enrol in industry led clinical trials. **c**: CT chest performed on a subgroup of participants

In total, 71 participants were recruited to the study (Fig. 1) and their baseline characteristics are outlined in Table 1. Pharmacy records were available for 44 (61.9%) participants and the mean adherence rate based on prescription pick-ups of LUM/IVA was 96.3%.

**Table 1:** Baseline Characteristics

	Participants (n = 71)		
Variable	n	Mean	Std Deviation
Age (years)	71	8.67	1.777
Sex			
Female, n(%)	71	36	50.7%
Height z-score	71	-0.13	1.015
Weight z-score	71	-0.15	0.889
BMI z-score	71	0.01	0.795
LCI_2.5_	28	9.15	2.412
ppFEV_1_	71	92.58	18.769
FEV_1_ Category			
< 40	71	1	1.4%
40-<70		6	8.5%
≥ 70		64	90.1%
Hospitalisations per annum	71	1.18	0.198
Pseudomonas status			
Clear	71	65	91.5%
Intermittent		5	7.0%
Chronic		1	1.4%

**Fig. 2:**
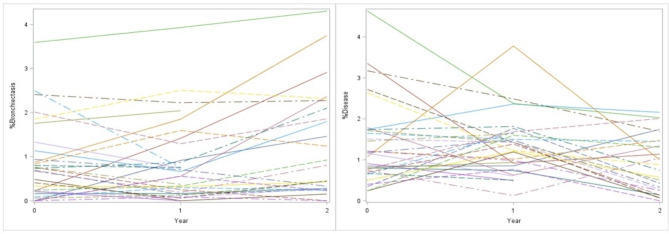
Spaghetti plots of change from baseline in %Bronchiectasis and %Disease over two years

**Fig. 3:**
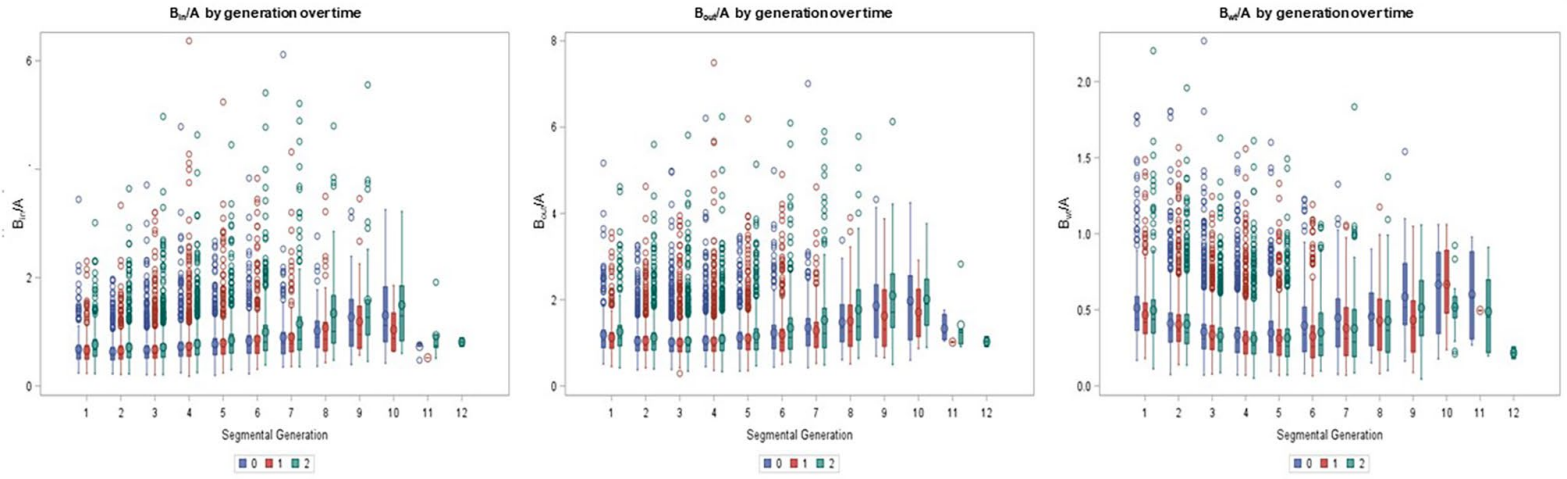
Boxplots of the BA ratios over two years. Each box shows median, interquartile range and outliers for each BA ratio at baseline, year one and year two

Of the 71 participants, 31 (43.6%) had ultra-low dose CT thorax at baseline, year one and year two (Table 2). There was a significant increase from baseline in the %Bronchiectasis PRAGMA-CF CT score over 2 years (0.82 to 1.24, p = 0.005) but no change in %Disease score (2.78 to 2.25, p = 0.138) (Fig. 2). There was a significant decrease in %Trapped air PRAGMA-CF score (0.13 to 0.07, p = 0.016) over two years but no change in %Mucus plugging (0.57 to 0.33, p = 0.221). Intra-observer and inter-observer reliability were excellent (> 0.8) for the two main PRAGMA CF scores %Disease and %Bronchiectasis (Table E2). There were significant increases in the B_in_/A-ratio over two years when examined in the first four generations and across all generations. There were no significant increases over any generations for B_out_/A-ratio and B_WT_/A-ratio (Fig. 3; Table 3).

**Table 2:** PRAGMA-CF CT scores at baseline and over two years of treatment with LUM/IVA.

	Baseline Mean (95% CI) n = 32		Year 1 Mean (95% CI) n = 32		Year 2 Mean (95% CI) n = 31		p-value
%Bronchiectasis	0.82	(0.48–1.16)	0.85	(0.51–1.19)	1.24	(0.89–1.60)	0.005*
%Disease	2.78	(2.12–3.45)	2.46	(1.79–3.14)	2.25	(1.55–2.94)	0.138
%Trapped Air	13.88	(9.34–16.42)	10.12	(6.52–13.71)	7.09	(3.29–10.89)	0.016*
%Mucous plugging	0.57	(0.31–0.83)	0.28	(0.01–0.54)	0.33	(0.04–0.62)	0.221

*p-values < 0.05. The p-value is for the trend over time (fixed effect of time) from the random coefficients model

**Table 3:** Changes in BA dimensions by segmental generation groups over study period

Segmental generation groups	B_out_/A ratio median (95% CI)		B_in_/A ratio median (95% CI)		B_WT_/A ratio median (95% CI)	
Median over all generations	0.010	(-0.015–0.035) p = 0.426	0.032	(0.012–0.050) p = 0.002*	-0.016	(-0.034–0.002) p = 0.079
Median over first four generations	0.010	(-0.0124–0.032) p = 0.381	0.029	(0.010–0.047) p = 0.003*	-0.014	(-0.031–0.003) p = 0.110

*p-values < 0.05. The p-value is from the test of the time effect in the repeated measures model

**Fig. 4:**
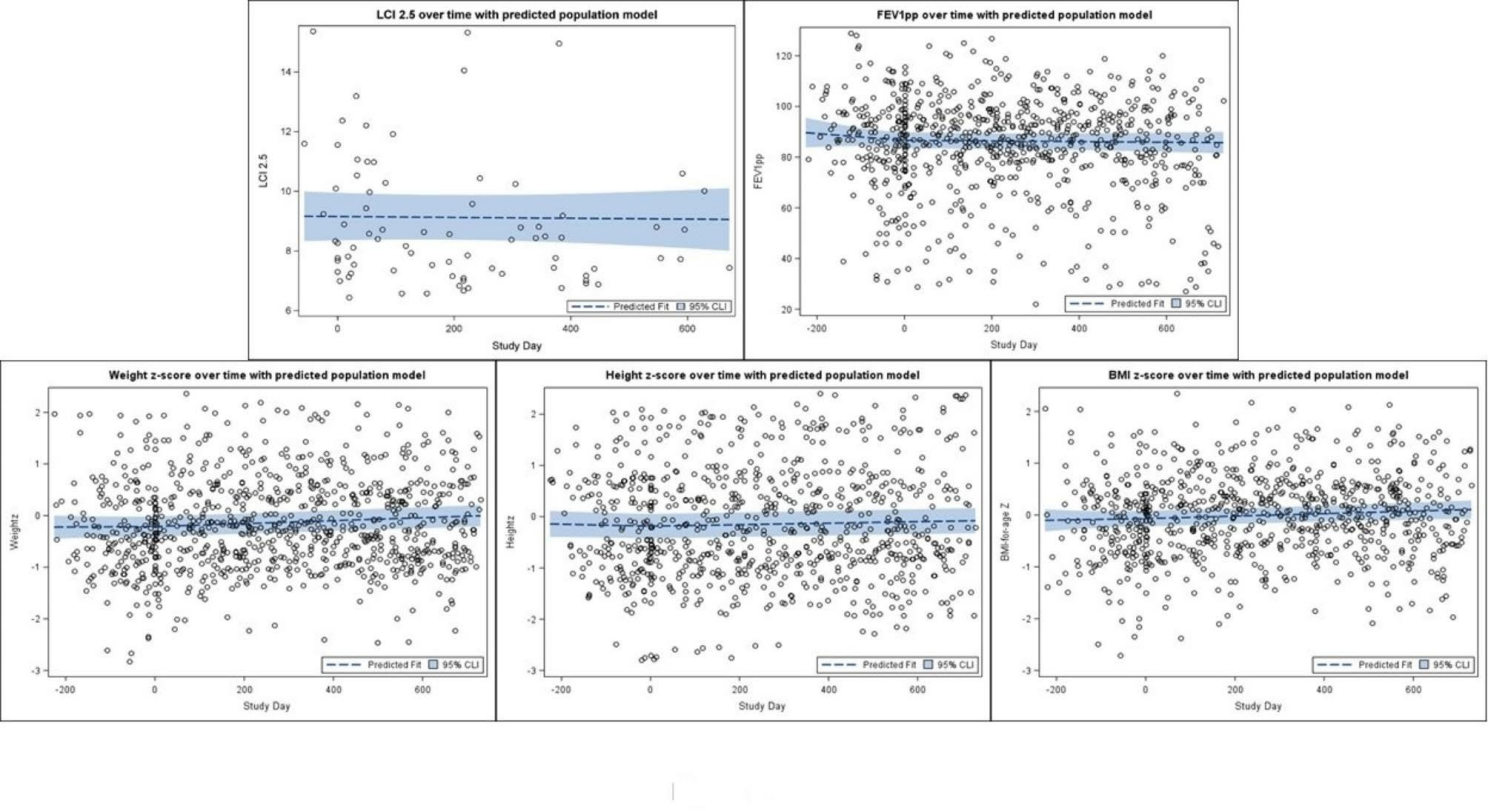
The data for all remaining clinical outcomes (LCI_2.5_, ppFEV_1,_ weight z-score, height z-score, BMI z-score) are presented as scatter plots along with the predicted modelled change in clinical outcomes over the study period. The change over time in clinical outcomes is expressed as an annualised slope, which is interpreted as the predicted annual change in the outcome. Study day was defined as the number of days pre/post initiation of LUM/IVA therapy

The data for all remaining clinical outcomes are presented as scatter plots along with the predicted modelled change in clinical outcomes over the study period in Fig. 4. The change over time in clinical outcomes is expressed as an annualised slope, which is interpreted as the predicted annual change in the outcome. Acceptable LCI measurements were collected on 28 (39.4%) participants at baseline. The mean annual change from baseline in LCI_2.5_ (annualised slope − 0.055 (95%CI -0.61 to 0.50), p = 0.85) measurements among participants were not significant. Regarding secondary clinical outcomes on the 71 participants, there was a significant change over time in BMI z-scores (0.09 (0.03 to 0.15), p = 0.005), weight z-scores (0.1 (0.06 to 0.15, p < 0.0001) and height z-scores (0.05 (0.02 to 0.09), p = 0.002) among participants. The mean annual change from baseline in ppFEV_1_ (-2.45 (-4.44 to 2.54), p = 0.66) measurements among participants was not significant.

The mean number of hospitalisations per annum was numerically lower after treatment with LUM/IVA, but this was not statistically significant (1.18 (0.85–1.65) versus 0.87 (0.59 to 1.28), p = 0.06).

## Discussion

In this study, while the degree of air trapping on CT imaging improved over the two years, bronchiectasis progressed and baseline abnormalities in the ratio of bronchial lumen to artery diameter worsened over the course of the study. We did not detect a significant change in LCI, likely due to the small number of measurements collected on participants. As expected, our study demonstrated improvements in nutritional and growth parameters in children aged 6–11 years with CF treated with LUM/IVA. Similar to the clinical trial in children of this age [20], we noted no significant change in FEV1.

Improvements in end organ function with LUM/IVA are modest compared to the improvements noted with previous (Ivacaftor in gating mutations) [27] and subsequent (Elexacaftor/Tezacaftor/Ivacaftor [ETI] for Phe508del) therapies, sometime referred to as ‘highly effective modulators’ in people with CF [28]. In many jurisdictions, ETI has replaced LUM/IVA as the modulator of choice in people with the Phe508del mutation; however, LUM/IVA is still in use in younger children and may remain in use in jurisdictions where approval or funding is not in place for other modulators. The data reported here remains relevant therefore and establishes some important information about the real-world use of modulators in children. While we demonstrated improvements in nutritional parameters, our study did not detect improvement in lung function and, despite the improvement in air trapping; bronchiectasis progressed in children treated with this modulator combination.

In relation to the impact of LUM/IVA on structural lung disease as measured by CT scores, there are no similar studies to date in children with CF aged 6–11 years. Real-world studies have demonstrated improvements in CT scores in association with Ivacaftor treatment in children aged six years or older with CF and the G511D mutation [29–31]. More recently, studies showed that CT scores improved following the introduction LUM/IVA in adolescents and adults [32–34]. Campredon et al. reported a significant decrease in mucus plugging and peribronchial thickening but not bronchiectasis using Bhalla CT scores in 283 adolescents and adults treated with LUM/IVA for one year [33]. A retrospective study of 34 adolescents and adults with CF described improvement in mucus plugging, but not any other outcome measures, using the Brody scoring system [34]. These studies did not employ spirometry control for image capture or standardisation of scanners to homogenise image quality. We used a validated spirometry controlled CT protocol [23] and the well-standardised PRAGMA CF scoring system [24] ensuring that our methods were sensitive for quantifying and monitoring structural lung changes [13]. The mean baseline %Disease in our cohort differed when compared with other studies examining children with CF in this age group (Table E3 and Figure E1) [35, 36]. Svedberg et al. retrospectively examined the rate of progression of structural lung disease scores in a comparable age cohort naïve to CFTR modulators. Although this study reported a higher baseline %Disease PRAGMA-CF score, they described a similar rate of yearly progression of %Disease as our study. Bouma et al. reported the progression of structural lung disease scores from preschool to school age in children with CF. This study had a lower baseline %Disease PRAGMA-CF score and a lower rate of annual progression compared with our study, but a younger age cohort was being studied. The differences seen between the different cohorts is most likely related to the different image acquisition methods used as well as varying clinical practices at different centres.

We did observe improvements in trapped air scores over two years, but despite this saw a progression of bronchiectasis. This may suggest that LUM/IVA has some effect in relieving small airway obstruction caused by inflammation in small airways but is unable to prevent the worsening of bronchiectasis caused by established or persistent infection and associated inflammation. In order to corroborate our findings, we examined BA dimensions throughout the segmental generations and found widening of the airways of our participants. This method has been shown to correlate well with bronchiectasis detected by PRAGMA CF scoring [17]. The increases in BA dimensions in respect to B_in_/A-ratio alone over two years, indicates an improvement in mucociliary clearance and thinning of the airway wall as described in a recent study [37]. The BA dimension findings correlate with the PRAGMA CT scores demonstrating that LUM/IVA had some effect on relieving small airway obstruction but the progression of structural lung damage in the form of bronchiectasis continued in our study participants. There is significant variation in the degree of structural lung disease in young children [15] and the clinical response to LUM/IVA in people with CF [38]. CFTR modulators have been shown to improve mucociliary clearance and are likely to improve air trapping [39]. Therefore, LUM/IVA may be effective at improving minor reversible structural lung disease, such as air trapping, but has little effect on more developed and irreversible changes such as bronchiectasis.

While clinical trials have reported improvements in pulmonary function with LUM/IVA in different age groups [7, 20]; there have been mixed findings in real-world settings. The PROSPECT study examined the impact of LUM/IVA in children aged six and above and adults [40]. In contrast to the clinical trials, they did not report improvement in ppFEV_1_, but did show an improvement of 0.55 units in LCI values at twelve months among 49 participants over six years of age [41]. Other real-world studies have reported improvements in ppFEV_1_ in those with impaired lung function at baseline but not in those with preserved lung function [42, 43]. In the French real-world study of outcomes with LUM/IVA, adolescents with impaired pulmonary function and raised LCI values (mean 12.3) did not demonstrate improvements in LCI or ppFEV_1_ over one year [44]. Similar to the clinical trials and real-world studies that included children aged 6–11 years; our study did not identify any improvement in ppFEV_1_. As this is a cohort of children with well-preserved lung function, our ability to detect significant changes in ppFEV_1_ was likely limited.

MBW measurements in our study did not detect any differences in LCI_2.5_ in children aged 6–11 years with CF over two years. The low numbers, natural variability in disease severity and relatively small effect size suggest that any effect of LUM/IVA has on ventilation inhomogeneity may be insufficient to be detected in a clinical population this size. Most participants only had MBW measurements taken at one or two time points at varying intervals after commencement of LUM/IVA rather than the prespecified four time points over two years as originally planned (Supplementary table S1). We conclude that the small sample size and low number of children with follow up measurements significantly hampered our ability to draw any assumptions on the lack of significant changes in LCI_2.5_ seen in our study.

Improvements in weight, height and BMI identified in this study are similar to those reported in clinical trials [20, 45] and real world studies examining the impact of LUM/IVA in the same age group [42]. Similarly, clinical trials demonstrated a decrease in exacerbation rates requiring IV antibiotics in adolescents and adults on LUM/IVA [7, 46, 47]. While this finding was replicated in one recent real-world study (8), several others failed to demonstrate a reduction in exacerbations requiring IV antibiotics in adolescents and adults [38, 42, 48]. The low number of exacerbations in our study, the relatively mild or early nature of lung disease of study participants and the relatively small number of participants may explain why we found no difference.

This study has a number of limitations. The COVID-19 pandemic, staff shortages and sub-optimal testing environment affected the collection of our MBW measurements in clinical settings. As this was an observational follow up study, we did not have a control group that would have strengthened our findings. In particular the absence of a CT control group means that we cannot determine the precise effect of LUM/IVA on structural lung disease, other than to say that while it may improve trapped air it does not appear to prevent the progression of bronchiectasis in this age group over two years. Only a small number of children were hospitalised for pulmonary exacerbations prior to or during the study period reflecting the clinical stability in most young children with CF and making detection of any treatment effect challenging. While the adherence rate with LUM/IVA was high in participants, we did not collect additional adherence data on other therapies. This may have impacted our findings, as children not adherent to airway clearance treatments are at an increased risk of developing bronchiectasis earlier. In addition, interpretation of FEV_1_ data would have been more robust if larger numbers were included. National registries would be better suited to analysis of data in relation to hospitalisations, FEV_1_ and other clinical data collected as part of routine care.

Notwithstanding its limitations, this is an important study. The demonstration of improvements in air trapping but worsening of bronchiectasis scores on LUM/IVA are original and underline the importance of collecting real world imaging data and the ongoing development and testing of more effective CFTR modulators. The negative findings in relation to lung function measures are perhaps not surprising in the context of the previous literature, the small number of participants in the study who had MBW measurements and the fact that the study was carried out on an unselected group of children in a real-world setting. The positive findings in relation to nutritional parameters corroborate other trial and real-world data and underline the clinical benefit of introduction of LUM/IVA in this age cohort. Ongoing work by our group will examine the subsequent impact of ETI on LCI, spirometry-controlled CT scores and other outcomes in this group of children and others as part of the RECOVER trial (NCT04602468).

### Electronic supplementary material

Below is the link to the electronic supplementary material.

Supplementary Material 1

## Data Availability

The datasets generated during and/or analysed during the current study are available from the corresponding author on reasonable request.
