# Supplementary material for: The clinical impact of Lumacaftor-Ivacaftor on structural lung disease and lung function in children aged 6–11 with cystic fibrosis in a real-world setting
Source: Respir Res. 2023 Aug 11;24:199. doi: 10.1186/s12931-023-02497-0 (PMC10416528; doi:10.1186/s12931-023-02497-0)
Supplement: Supplementary file 1 — Supplementary Material 1 [file 12931_2023_2497_MOESM1_ESM.docx]

**Supplementary materials**

**Table E1: LCI_2.5_ follow up measurements.** LCI data was not linearly collected on participants every six months as intended due to the COVID-19 pandemic. Thus, LCI measurements were taken within three months of LUM/IVA initiation and again at random intervals over the two-year study period.

| **Number of LCI_2.5_ per patient** | **Total n=28** |
| --- | --- |
| **1** | 10 |
| **2** | 5 |
| **3** | 8 |
| **4** | 3 |
| **5** | 2 |
| **6** | 2 |

**Table E2: Correlation coefficients for intra and inter observer agreement**


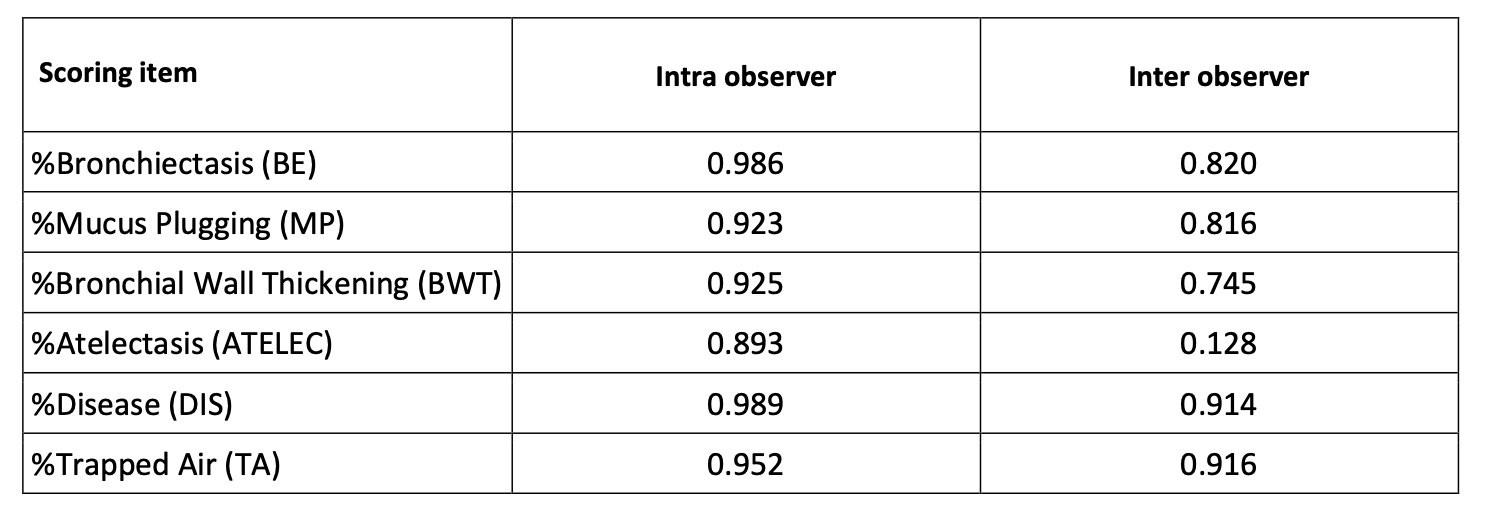


**Table E3: Comparison of % disease at baseline in similar cohorts**

| **Cohort** | **Sample size** | **Mean age, years (range)** | **Baseline % disease (95% CI)** |
| --- | --- | --- | --- |
| **CFORMS** | 32 | 8.8 (6.05 – 11.56) | 2.78 (2.12 - 3.45) |
| **Bouma et al. 2020** | 61 | 10.65 (8.75 - 11.83) | 1.73 (0.28 - 2.86) |
| **Svedberg et al. 2020** | 75 | 6.8 (6-12) | 4.67 (0.30–17.49) |

**Figure E1: Stacked histogram sorted by % disease at baseline**

(BE = bronchiectasis, Plug = mucus plugging, AWT = airway wall thickness, Atelec = atelectasis)
